# Correction to: ‘Social selection within aggregative multicellular development drives morphological evolution’ (2021) by La Fortezza and Velicer

**DOI:** 10.1098/rspb.2022.2290

**Published:** 2022-12-21

**Authors:** M. La Fortezza, G. J. Velicer

*Proc. R. Soc. B* **288**, 20211522. (Published online 24 November 2021) (https://doi.org/10.1098/rspb.2021.1522)

Corrected text (in bold):

Funding. This work was funded in part by an EMBO Long-Term Fellowship (ALTF 1208-2017) to M.L.F. and **SNSF grant no. 310030B_182830 to G.J.V**.

This has been corrected on the publisher's website.

